# Escalation and De-Escalation of Adjuvant Radiotherapy in Early Breast Cancer: Strategies for Risk-Adapted Optimization

**DOI:** 10.3390/cancers16172946

**Published:** 2024-08-23

**Authors:** Guenther Gruber

**Affiliations:** 1 Institute for Radiotherapy, Klinik Hirslanden, Witellikerstrasse 40, CH-8032 Zurich, Switzerland; guenther.gruber@hirslanden.ch; 2 Medical School, University of Nicosia, CY-1700 Nicosia, Cyprus; 3 Medical Faculty, University of Berne, CH-3000 Berne, Switzerland

**Keywords:** adjuvant radiotherapy, omission of radiotherapy, partial breast irradiation, de-escalation, escalation, loco-regional irradiation, optimization

## Abstract

**Simple Summary:**

Radiotherapy (RT) is a cornerstone in the adjuvant treatment of breast cancer. Continuous technical improvements allow better sparing of organs at risk compared to the past with a potential reduction of RT-related toxicity. Whereas prior trials focused on histopathological criteria, mainly T- and N-stage, biological parameters like endocrine responsiveness and proliferation helped to identify a low-risk subgroup in which omission of RT is an option. Ongoing trials are incorporating molecular markers and the response to neoadjuvant systemic therapy for additional risk stratification. De-escalation regarding volume (partial breast irradiation only—PBI) can be used in selected cases. Hypofractionated regimens should be standard. In contrast, the omission of axillary dissection in node-positive disease led to an escalation of regional RT, and RT for oligometastatic disease is becoming increasingly popular. Studies are ongoing to test if any axillary treatment can be omitted and which oligometastatic patients do really benefit from RT.

**Abstract:**

Postoperative radiotherapy (RT) is recommended after breast-conserving surgery and mastectomy (with risk factors). Consideration of pros and cons, including potential side effects, demands the optimization of adjuvant RT and a risk-adapted approach. There is clear de-escalation in fractionation—hypofractionation should be considered standard. For selected low-risk situations, PBI only or even the omission of RT might be appropriate. In contrast, tendencies toward escalating RT are obvious. Preoperative RT seems attractive for patients in whom breast reconstruction is planned or for defining the tumor location more precisely with the potential of giving ablative doses. Dose escalation by a (simultaneous integrated) boost or the combination with new compounds/systemic treatments may increase antitumor efficacy but also toxicity. Despite low evidence, RT for oligometastatic disease is becoming increasingly popular. The omission of axillary dissection in node-positive disease led to an escalation of regional RT. Studies are ongoing to test if any axillary treatment can be omitted and which oligometastatic patients do really benefit from RT. Besides technical improvements, the incorporation of molecular risk profiles and also the response to neoadjuvant systemic therapy have the potential to optimize the decision-making concerning if and how local and/or regional RT should be administered.

## 1. Introduction

Adjuvant treatment in early breast cancer is based on its multi-disciplinarity and is becoming more and more complex. Radiotherapy (RT) either after breast-conserving surgery (BCS) or mastectomy (PMRT) has a long tradition and is an integral part in the treatment algorithm, but the era of ‘one-size-fits-all’ treatments is behind us. The tumor situation and the risk of relapse can be characterized more precisely compared to previous years. Optimization of the treatment approach either as escalation or de-escalation is key.

Undoubtedly, local tumor control will be significantly improved by adjuvant RT [1]. This is widely independent from the subgroup of patients with an average 3- to 4-fold relative risk reduction [1]. This improvement in local/regional control may also convert into better overall survival (OS). However, the absolute risk reduction is mainly defined by the risk at diagnosis, and the gain in local control in low-risk patients might be only a few percent. In addition, impact on OS in low-risk patients has not been proven or is presumably in a range which is considered clinically negligible.

Furthermore, no positive effect exists without side effects. We have to be aware of radiotherapeutic acute and potential long-term toxicity. Nearly all patients will have local inflammatory reactions towards the end of irradiation, which are in general well manageable and seldom severe. A lot of different interventions have been tried to reduce acute toxicity for the skin; one of the most promising might be the use of Mepithel film, which could reduce the incidence of radiation dermatitis and improved patient-reported outcomes (PROs) in a recent meta-analysis of three randomized controlled trials [2]. Using modern techniques with intensity-modulated fields led to the reduction of acute toxicity. In daily practice, more pronounced (exudative) skin reactions are uncommon and recover normally within a few weeks. Late reactions are more important, as late reactions often represent a scarring of tissue. Regarding local RT, severe induration of the breast is an issue, which can finally lead to the removal of the breast in rare cases. The loss of reconstruction, especially after implant-based approaches, is a major concern for PMRT, as well as the risk for persistent arm lymph edema after regional irradiation especially in patients who underwent additional axillary lymph node dissection (ALND). Interestingly, limited data could not demonstrate a statistically significant impact on quality of life either in the low-risk situation (5 yrs of data from the PRIME 1 study) [3] or in the postmastectomy setting (2 yrs of data from the SUPREMO trial) [4]. Increasing implementation of PRO data in clinical studies and in daily routine will give us more insights regarding this important issue. Last but not least, some EV toxicity for the lungs, heart and ribs and the possibility of tumor induction by ionizing irradiation demand the optimization of adjuvant RT and a risk-adapted approach.

In the present manuscript, important topics regarding de-escalation and also escalation will be identified, and some strategies are discussed to optimize the decision-making regarding if and how local and/or regional RT should be administered. The selection of topics has no claim to completeness and reflects the personal choice of the author.

## 2. Possibilities of De-Escalation (See Figure 1)

### 2.1. ‘Sophisticated’ RT

Intensity-modulated RT (IMRT) and volumetric-modulated arc therapy (VMAT) allow an optimized dose distribution for the target volume and dose application. On-board imaging (OBI), including cone-beam CTs (CBCTs), is of great help for appropriate patient positioning. Acute toxicity like exudative skin alterations will be reduced substantially [5,6]. Irradiation in prone position or breath-controlled in deep inspiration can better spare the lungs and heart, and especially in patients with large breasts irradiation in prone position can reduce acute reactions [7,8,9]. In general, photons are used. Few centers have the possibility of proton treatment, which is quite complex and expensive due to several reasons. For example, proton treatment in Switzerland is only available at the Paul Scherrer Institute (PSI) in Villigen, Aargau, for certain indications, but breast cancer treatment will not be reimbursed by insurance companies, which increases the burden to use protons in a broader application. According to the database of the Particle Therapy Cooperative Group (PTCOG; www.ptcog.site; accessed on 29 June 2024), a total of 350,336 patients have been treated with protons worldwide from 1954 to 2023, about 7% of them for breast cancer. Protons have a special dose distribution in tissue and might be beneficial in situations where an organ at risk is just in the nearby area of the target volume, which can be an issue for the heart, if loco-regional RT including the parasternal lymph nodes is indicated in left-sided breast cancer. A pragmatic trial in the US (RadComp; RTOG 3510; NCT02603341) [10] is evaluating the comparison of photon and proton RT. The primary endpoint is major cardiovascular events. The first results will not be available before 2032. The current evidence, as well as ongoing randomized trials, are perfectly summarized in a systematic review and meta-analysis by Holt et al.; a total of 32 non-randomized studies with 1452 patients and a follow-up of 2–59 months (mo) were included [11].

**Figure 1:**
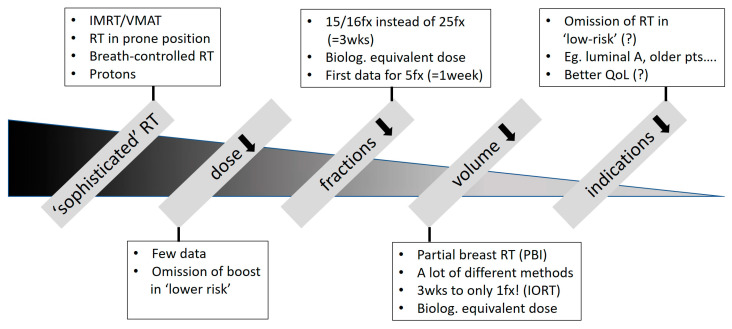
Possibilities for RT de-escalation. Abbreviations: IMRT = intensity-modulated radiotherapy; VMAT = volumetric-modulated arc therapy; RT = radiotherapy; fx = fraction; pts = patients; QoL = quality of life; wks = weeks; IORT = intraoperative radiotherapy.

The most important point is an intra- and interobserver variability in contouring the planning target volume (PTV) despite contouring guidelines, e.g., from ESTRO [12]. Artificial intelligence can help, and several systems for automated contouring are on the market. Not only regarding contouring but also in treatment planning, an inter-planner variation is evident [13]. Knowledge-based treatment planning has the potential to produce plans of uniform quality by reducing the inter-planner variability and the duration of the optimization process [14].

### 2.2. Dose Reduction

After demonstrating that boost irradiation can decrease local failure rates [15], the interest in conducting trials regarding dose reduction was low. To my knowledge, the St. Georg and Wollongong breast boost trial has never been published regarding local control [16]. This trial randomized 688 patients to the control arm of 50 Gy in twenty-five fractions and the boost arm of 45 Gy in twenty-five fractions to the whole breast followed by a sixteen Gy in an eight fraction electron boost [16]. A small whole-breast dose reduction outside of the tumor bed (from 40 Gray—Gy to 36 Gy in 15 fractions) was not inferior in the IMPORT LOW trial [17]. In addition, compared to previous times, boost irradiation is generally no longer given to patients at lower risk considering the pros and cons, as a very small advantage in local control might be outweighed by an increase in severe breast fibrosis [18].

### 2.3. Reduction in Fractions

Hypofractionated RT schemes use higher single doses, which is biologically more effective. As a consequence, total dose and overall treatment time have to be reduced to reach comparable biological dose effects. Over the decades, 25 fractions with a single dose of 2 Gy in total of 5 weeks were considered standard, which has been replaced by overwhelming data regarding a three-week treatment of 15 or 16 fractions up to a total dose of 40 to 42.5 Gy in breast conservation, for example, the START A and B trials [19], and after mastectomy, like the Chinese randomized phase 3 trial by Wang et al. [20]. There is a clear trend to further reduce the fraction number and overall treatment time. The FAST FORWARD fractionation scheme of five fractions of 5.2 Gy in a single dose (ultrahypofractionation) has been already widely adopted, especially during the COVID pandemic in several countries, despite the fact that only five-year data have been published (but with excellent results) [21]. In this trial, patients were allocated to either 40 Gy in fifteen fractions (over 3 weeks), 27 Gy in five fractions (over 1 week), or 26 Gy in five fractions (over 1 week) to the whole breast or chest wall, and the arm with 26 Gy in five fractions over 1 week was non-inferior to the standard of 40 Gy in 15 fractions over 3 weeks in regard to local tumor control and normal tissue effects [21]. A German expert panel (DEGRO) is considering the 3-week schedule standard still, as moderate or marked late effects increased with longer follow-up in disadvantage of the ultrahypofractionated arm for most items [22]. There are not many data on ultrahypofractionation for regional node irradiation; results for the nodal subgroup in the FAST FORWARD study are pending. Both simple and complex RT techniques are allowed in the ongoing HYPART trial [23] to create the possibility for low-income countries to also follow this approach. The ESTRO Advisory Committee in Radiation Oncology Practice state that moderate hypofractionation (the 3-week scheme) can be offered to any patient for the whole breast, chest wall (with or without reconstruction), and nodal volumes; the five-fraction schedule can also be offered for non-nodal breast or chest wall (without reconstruction) RT either as standard of care or within a randomized trial or prospective cohort [24].

### 2.4. Volume Reduction—Partial Breast Irradiation Only (PBI)

As most of the in-breast failures occur in the proximity of the initial tumor site, several studies have been launched to test the irradiation of the initial tumor bed plus a safety margin of 1.5 to 2 cm instead of whole breast irradiation. Reducing RT volume allows an increase in the single dose and consequentially also a reduction in fraction number and overall treatment duration. There are several different treatment modalities and schemes available, and PBI can be given pre-, intra- or postoperatively. In general, patients with low risk for failure have been included in such trials. Both 5 yr and 10 yr outcome data tend to be comparable and low in regard to local failures in most studies also with reduced toxicity for the PBI arm. For intraoperative treatment (IORT) with a single high-dose fraction (about 20 Gy), local failure rates are significantly higher in the ELIOT trial [25]. Another IORT trial (TARGIT-A) yielded similar outcome data: the non-breast-cancer-specific mortality was even better with IORT [26] but was criticized mainly due to statistical issues. Overall, mortality seems to be comparable, as the heart death rate is 0.3% less for PBI [27]. It is important to allow the normal tissue enough recovery time. Although less acute toxicity has been observed, there was an increase in moderate late toxicity and worse cosmesis, which might be related to the twice-per-day treatment scheme (10 fractions of 3.85 Gy over 5–8 days) in the RAPID trial [28]. For low-risk patients, PBI is well accepted in several guidelines, e.g., the German S3 guideline [18] or the current NCCN guideline [29].

### 2.5. Reduction of Indication (=Omission of RT)

The smaller the initial risk of failure, the less is the potential absolute gain due to adjuvant RT. Many trials have evaluated the omission of RT in low-risk situations. Commonly used criteria for low risk in recent trials after BCS are age (above 65 or 70 years), T-stage (T1 or small T2), hormone responsiveness, node-negative disease, and no G3 differentiation. All trials have shown the benefit of additional RT regarding local control. However, the absolute difference was rather small, and there was no impact on cause-specific or overall survival. Recently the 10 yr results of the PRIME 2 study have been published [30]: In selected lower-risk patients, to whom adjuvant antihormonal therapy has been prescribed, local failure rates at 10 years were 0.9% with versus 9.5% without RT, with similar OS in both arms. Especially in older women, the omission of RT is an option. In discussing the pros and cons of additional RT with the patient, it is important to mention that these excellent data in regard to local control and survival have been achieved in the backbone of antihormonal therapy. In daily practice, it is not seldom the case that patients at lower risk are reluctant to take five years of tamoxifen or aromatase inhibitors. As a consequence, they often choose RT as their only treatment. Additional parameters would be helpful to redefine risk and optimize adjuvant treatment for these patients.

There is a long-lasting discussion about PMRT, especially in the intermediate risk group (T1-2 N1). Since the publication of the Danish trials in 1997 and 1999 [31,32], PMRT is recommended for larger T-stage tumors (pT3/pT4) or patients with four or more involved axillary lymph nodes. The 30-year data demonstrate that optimal long-term treatment benefit can only be achieved in both optimal loco-regional and systemic tumor control. Furthermore, PMRT did not result in excess ischemic heart damage, nor in other non-breast-cancer-related deaths [33]. Due to some criticism of the DBCG 82b/c trials regarding the quality of surgery and systemic therapy, no clear indication for PMRT has been given in the subgroup of patients with pT1/pT2 and 1–3 positive nodes, but the NCCN guidelines state that additional RT ‘should be strongly considered’ [29]. Interestingly, this did not change over more than 20 years. Often, additional risk factors are used to decide about PMRT, ‘yes’ or ‘no’ [18]. The results of the SUPREMO trial, in which patients with 1–3 positive nodes, with stage pT2pN0 and the presence of grade 3 and/or vascular invasion and with stage pT3pN0 were randomized between loco-regional or no RT following mastectomy, are pending [34]. As ALND is nowadays seldom performed, the exact number of lymph nodes is unknown in most patients. Other factors besides number of nodes are urgently needed to optimize the indication for PMRT.

## 3. Possibilities of Escalation (See Figure 2)

### 3.1. Preoperative RT

RT as soon as possible might be beneficial [35]. A large randomized trial has been launched in Germany to test this approach (Ref. [36], NEORAD trial); the first patient was randomized just recently in March 2024. This trial is a multicenter randomized phase 3 trial investigating whether preoperative RT improves disease-free survival compared to postoperative RT after neoadjuvant chemotherapy in patients with breast cancer at high risk of recurrence. A total of 1826 patients are planned. Optimal reconstruction strategies in the setting of PMRT are challenging [37]. Especially for patients for whom mastectomy and breast reconstruction are planned, preoperative RT would avoid irradiation of a reconstructed breast. This might lead to fewer side effects and better cosmesis. At ASTRO, 2022 retrospective data have been presented in patients with mastectomy and DIEP reconstruction, either with pre- or postoperative irradiation [38]. The authors observed a significantly higher incidence of flap contracture (41.9% vs. 1.9%) and fat necrosis (19.4% vs. 12.9%) in patients with PMRT compared to preoperative RT. A good/excellent cosmetic outcome was 96.1% in women with preoperative RT vs. 80.6% with PMRT [38]. Preoperative RT might also be beneficial for defining exact tumor location for PBI only or as a preceding boost, possibly to achieve higher pCR rates [39,40].

**Figure 2:**
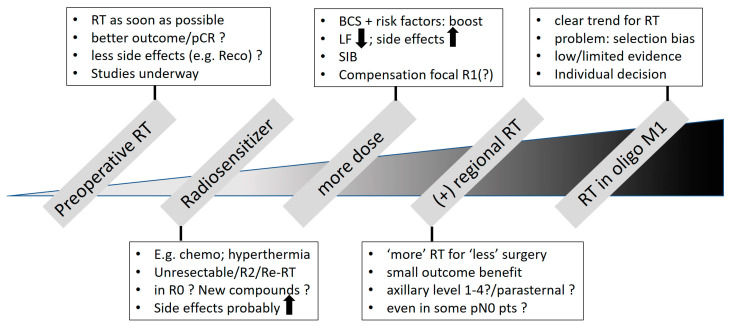
Possibilities for RT escalation. Abbreviations: RT = radiotherapy; pCR = pathological complete remission; reco = breast reconstruction; chemo = chemotherapy; Re-RT = re-irradiation; BCS = breast-conserving surgery; LF = local failure; SIB = simultaneous integrated boost; pts = patients; oligoM1 = oligometastatic disease.

### 3.2. Radiosensitizer

Chemotherapy or hyperthermia given simultaneously with RT are well-known radiosensitizers. They might be used especially in situations with unresectable tumor or local relapse and previous RT to optimize local control [41]. Concomitant chemotherapy can increase relevant side effects to organs at risk, like the heart. In a study of 660 consecutive patients concomitant left-side RT with doxorubicin dose ≥ 250 mg/m^2^ and hypertension were independent risk factors for cardiovascular events [42]. If both treatments are given, standard of care is a sequential approach with chemotherapy first. Preoperative chemotherapy is becoming more and more popular, and, as stated above, there is also some rationale for preoperative RT. Therefore, the combination of both in the preoperative setting receives more attention. An excellent overview has been recently published [43]. Three studies [44,45,46] plan to analyze the combination of RT and immunotherapy. Novel drugs, also in combination, are rapidly entering clinical practice. However, data about their optimal use with RT are seldom provided. An international multidisciplinary consensus summarized this topic, and recommendations have been recently published [47]. A very recent multicenter retrospective study suggests that concurrent use of antibody–drug conjugates and brain irradiation is associated with a higher risk of symptomatic radiation necrosis (27% vs. 7% cumulative incidence at 2 years; *p* = 0.014) in HER2-positive breast cancer patients [48].

### 3.3. Dose Escalation (Boost)

An additional dose to the tumor bed (= boost) can improve efficacy after BCS. Besides the possibility of decreasing local failure rates (relative risk reduction of about 50%), no impact on OS could be demonstrated, and giving more doses also has results in an increase in side effects [15,49]. Therefore, boost irradiation is only recommended in the presence of risk factors. Some of them are young age, node positivity or close margins. In the current German S3 guideline, a boost is indicated in patients up to 50 years old or in G3/>T1/triple negative or Her2 positive tumors [18]. Especially young patients may benefit from a high-dose boost. This has been tested in a large phase 3 trial with 2421 patients who were all aged 50 years or younger, randomized to a standard dose boost (16 Gy in eight fractions of a 2 Gy single dose) or a high-dose boost (26 Gy in thirteen fractions). The 10 yr results were presented at the 14th European Breast Cancer Conference in March 2024 [50]. The 10 yr local failure rate was 4.4% with the 16 Gy boost vs. 2.8% with 26 Gy. According to the authors, this small benefit (1.6% better local control) does not justify the increased impact on cosmetic outcome, as 48% of patients with the 26 Gy boost experienced severe or moderate fibrosis vs. 27% with 16 Gy [50].

Integrating the boost simultaneously (SIB) to whole-breast RT provides better dose homogeneity and reduces overall treatment time. According to a systematic review in 2022 of nine published trials, one of them randomized, an SIB with a standard dose seems to be effective and safe [51]. This has been recently confirmed by the IMPORT HIGH trial, whereas a further boost-dose escalation from 48 to 53 Gy was not beneficial [52]. In this phase 3 trial, 2617 patients with pT1-3pN0-3aM0 breast cancer and BCS were randomized to one of three treatment groups: The control group received 40 Gy in fifteen fractions to the whole breast and a sequential tumor-bed boost of 16 Gy in eight fractions. Test group 1 received 36 Gy in 15 fractions to the whole breast, 40 Gy in 15 fractions to the partial breast, and 48 Gy in 15 fractions as a concomitant boost to the tumor bed. Test group 2 received 36 Gy in 15 fractions to the whole breast, 40 Gy in 15 fractions to the partial breast, and 53 Gy in 15 fractions as a concomitant boost to the tumor bed [52]. Results from the German HYPOSIB trial with a total of 2324 patients are eagerly awaited [53]. In the experimental arm of the HYPOSIB trial, patients received hypofractionated WBRT with 40 Gy in 16 fractions of 2.5 Gy with an additional SIB of 0.5 Gy to the tumor bed, resulting in a total dose of 48 Gy to the tumor bed [53]. Preliminary safety data have been presented at the Annual Meeting of the American Society for Radiation Oncology (ASTRO). Acute skin reactions were less pronounced and occurred two weeks earlier in the HYPOSIB arm than in the control arm [53]. The Radiation Therapy Oncology Group (RTOG) 1005 trial investigated 48 Gy SIB in 15 fractions with noninferiority for local control and similar toxicity for SIB and the sequential boost [54]. First data have been presented also for the five-fraction scheme [55], and other trials are ongoing [56,57]. The HYPORT trial is testing the five-fraction scheme; both SIB and sequential boost techniques are allowed [56], and the RHEAL trial is using sequential boosts [57]. The TARGIT-B trial testing an intraoperative versus a sequential boost is still ongoing; the data lock will be the end of October 2024 [58]. Interestingly, according to the Dutch database, boost irradiation might counteract the risk of focally involved resection margins (R1), with a small difference in local failure rates at 5 years (1% with vs. 3% without re-excision) but without impact on DFS or OS [59].

### 3.4. Additional Regional RT

Regional lymph node irradiation resulted in a small but significant improvement in cause-specific survival (CSS) and OS in newer trials. A significant reduction in breast cancer mortality was even seen in pN0 patients, but the difference was very small—1.6% at 15 years [60,61]. It is unclear which lymph node area RT (axilla/periclavicular/parasternal) counted most. Newer data indicate that RT of the parasternal lymph nodes might be beneficial, especially in medially located, node-positive tumors and with the modern RT technique used [62,63,64]. In a retrospective analysis of the EORTC 22922/10925 randomized trial, the 15 yr OS benefit was 4.9% with the ‘modern’ vs. 1.1% with the ‘old’ technique [64]. Especially trials like ACOSOG Z0011 [65], AMAROS [66] and OTOASOR [67], in which sentinel LN positive (SN+) patients were randomized either to ALND or SLND alone, have led to a dramatic decrease in ALND in patients with positive sentinel lymph node macrometastasis/es in favor of regional RT (identical tumor control, and ca. 50% less arm lymph edema). Further optimization is needed to define the subgroup of node-positive patients in which any axillary treatment can be safely omitted.

### 3.5. RT in Oligometastatic Situations

The concept of an optimized local tumor control in oligometastatic disease is currently being evaluated in multiple cancer types, including breast. Due to the high likelihood of a selection bias, it is unclear which subgroup of patients with oligoM1 might benefit [68]. The randomized NRG-BR002 trial has been presented at the Annual Meeting of the American Society of Clinical Oncology (ASCO) 2022 [69]: In this trial, women with pathologically confirmed metastatic breast cancer to ≤4 sites and a controlled primary tumor were eligible. A total of 129 patients were randomized (1:1) to standard-of-care systemic treatment (SOC) with or without stereotactic body RT and/or surgical resection of all metastases. The addition of metastasis-directed therapy to SOC systemic treatment did not show a signal for better progression-free survival or OS; therefore the trial did not proceed to the preplanned phase 3 component. More data are needed to answer this important question. The German S3 guideline recommends an individual and multidisciplinary decision in selected cases [18]. An excellent overview of ongoing clinical trials of metastasis-directed therapy for oligoM1 is provided by Merloni et al.; at least nine studies are active/recruiting [70].

## 4. Possibilities for Optimization

### 4.1. Technical Issues

As mentioned above, a lot of technical improvements have led to the possibility of very sophisticated RT planning and application. Intensity-modulated RT (IMRT); volumetric-modulated arc therapy (VMAT); image-guided positioning of the patient and planning target volume (PTV); prone positioning, especially for large breasts; and breath-controlled RT (deep inspiration), especially for left-sided breast cancer, are becoming standard of care in RT departments worldwide. The basis for the whole treatment chain is the contouring of structures which should be irradiated (e.g., PTV) or spared (e.g., organs at risk). However, there is a well-known inter-observer variability in contouring. We have tested three different artificial intelligence (AI) solutions for contouring in our department; two of them were considered excellent to counteract the inter-observer variability. MRI has a superior soft-tissue contrast compared to standard computer tomography (CT)-based planning. For the definition of the tumor bed (e.g., for PBI as boost or as sole irradiation), MRI-based planning might be beneficial. Furthermore, MR-Linacs allow for online adaptive treatment planning before each fraction [71,72], but the impact of this approach regarding clinical benefit is not well known. As the patient’s time on the treatment couch is clearly increased, so far only few institutes (to our knowledge, e.g., only one in Switzerland) have installed an MR-Linac, and even fewer are using this machine for breast cancer treatment. Proton treatment is of emerging interest, but results from at least six ongoing randomized trials in Denmark, the UK, Thailand and the USA have to be awaited [11]. In general, a potential therapeutic gain due to further technical improvements in the future seems to be becoming smaller and smaller.

### 4.2. Biology-Based Optimization

Several important questions regarding more or less RT are unanswered. While biology- and molecular-driven decisions about adjuvant chemotherapy are current standard, there is lack of data for RT. In low-risk situations, it is still unclear in which subgroup RT can be safely omitted. The individual randomized trials showed a significant increase in local failures without RT but no impact on OS. In a meta-analysis, omission of RT led to a 6.8-fold increased local failure risk (hazard ratio (HR) 6.8, 95% confidence interval (CI) 4.23–10.93) [73]. According to results from retrospective analyses, molecular signatures have the potential to select patients for RT omission [74]. However, these tests should be evaluated prospectively before they are used in routine clinical practice. Biology-driven prospective studies including molecular classifiers are ongoing (see Table 1). Nearly all trials have antihormonal treatment as their backbone, whereas adjuvant RT is omitted either in single-arm trials or in a randomized fashion. The first results with two to five years follow-up revealed excellent results so far; however, long-term data are needed. I am co-chair of the ongoing EXPERT trial [75]. This trial is testing the omission of whole-breast RT in low-risk patients defined by clinical/histopathological parameters and the PAM50 assay. I have to admit that randomizing patients into the trial is not easy. Two main reasons are fear of undertreatment and a must to undergo endocrine therapy (ET). In a patient survey, ET had the biggest negative impact on QoL, and patients would rather choose RT instead of ET [76]. About half of the patients were 75 years or older. If patients are low-risk, it is questionable if patients really need both RT and ET. In a retrospective analysis of 496 patients with a median age of 76 years, long-term recurrence rates were extremely low, even with the omission of endocrine treatment [77]. In the phase 3 EUROPA trial, patients aged 70+ years will be randomized to either RT or ET alone. The primary endpoint will be health-related quality of life at 2 years [78]. Another approach to define low risk is the integration of preoperative MRI to assess low local tumor burden. Women aged 50 years or older with cT1N0 non-triple-negative breast cancer were eligible. Those with unifocal cancer had BCS and adequate systemic treatment. If pT1N0/N1mi, RT was omitted (*n* = 201) [79], the invasive local failure rate at 5 years was 1.0% (upper 95% CI 5·4%) [79]. As local failure rates may increase, especially after the end of adjuvant systemic therapy, long-term follow-up has to be awaited to define subgroups of patients who can safely forego RT.

For the distinction of no RT, local or loco-regional RT, mainly T- and N-stage are used. Especially the appropriate treatment of the axilla by surgery and/or RT have been discussed heavily over the last years. The abovementioned sentinel trials [65,66,67] had as a consequence that the exact number of axillary lymph nodes is not known any longer due to the omission of ALND. Whereas AMAROS [65] and OTOASOR [67] used large comprehensive RT fields, ACOSOG Z0011 [65] has furthermore ignited the discussion about the necessity of regional irradiation in these patients, as according to the treatment protocol, only breast RT was required. However, the study was lousy in regard to RT documentation. For 605 of 856 patients, no RT info was given, and a detailed RT record review was only possible for 228 patients [86]. According to the review, about 81% received tangential RT alone, about half of them with high tangents encompassing parts of the lower axilla. Some patients even received directed nodal irradiation via a third field. Overall, there was no significant difference between treatment arms in the use of protocol-prohibited nodal fields [86]. As it is common practice to provide treatment plans (e.g., in case of re-irradiation), it is somehow strange that a detailed review was possible for only 228 patients. My personal hypothesis is that the local radiation oncologists did not know about the field restriction within the trial and have not sent the documents due to realizing this after request. Indeed, the radiation oncologists’ use of differing radiation fields in this trial was associated with treating higher-risk patients defined by nomograms [87]. The authors observed a significant association with higher nomogram estimated risk and supraclavicular irradiation but not high tangents [87]. Uncertainties in the ACOSOG Z0011 trial regarding RT led to the discussion of intentional (INT) versus incidental (INC) RT of the axilla and to the implementation of RT quality assurance (QA) programs in trials dealing with optimal axillary treatment. The TAXIS trial randomizes patients with clinically node-positive disease either to ALND or loco-regional RT after sentinel and targeted axillary surgery. In both treatment arms, a comprehensive QA program should give important information concerning INT vs. INC irradiation of the axilla [88]. The OPTIMAL phase 3 trial randomized 442 patients to either incidental (by breast only RT) or intentional regional RT [89]. With a median follow-up of 3.7 years, the estimated DFSs at 5 years were 93.7% and 93.8% in the INC and INT irradiation groups, respectively [89]. Neither in the recently published SINODAR ONE [90] and SENOMAC [91] randomized trials nor in the BOOG 2013-07 nationwide registry trial [92] are exact RT doses to the axillary regions provided. All of them support the omission of ALND after a positive sentinel lymph node biopsy [90,91,92]. Thus, the optimal adjuvant regional node irradiation volume for early-stage breast cancer with T1-2N1 remains undetermined. The T-REX phase 3 study randomizes patients with ER+, Her2 negative T1-2 disease with 1–2 sentinel macrometastases (mets) to regional RT or not [93]. The accompanying translational protocol aims to decipher the prognostic and predictive role of tumor biology and gene expression analysis for their potential integration into future adjuvant RT individualization [93]. The most important ongoing trial is probably the Tailor RT (MA39) trial [94], as it incorporates tumor biology (including Oncotype DX^®^ recurrence score—RS) as an eligibility criterion. It was initially started for patients with the following criteria: low Oncotype Dx RS < 18; ER positive/Her2 negative; pT1-2; age 40+ years; and 1–3 macromets after ALND/1-2 SN macromets after BCS/1 macromet after mastectomy, amendments allow also for randomization of patients with micromets, 2 SN macromets after mastectomy, T3N0 disease, age limit 35+ years and an Oncotype DX RS up to 25. A total of 2140 patients will receive loco-regional RT as standard vs. breast RT only after BCS and no RT at all after mastectomy [94]. A much smaller randomized phase 2 trial, the IMNI PRECISION trial, uses a ‘low-risk’ genomic score (RecurIndex^®^ test) to randomize 214 patients, who are clinically at higher risk, to internal mammary node irradiation or not [95]. A large SEER database study (*n* = 6509) highlights the possible role of the RS in predicting the outcome of adjuvant RT in T1-2N1 luminal BC patients undergoing BCS. Adjuvant RT was not associated with better 5-year outcomes in the low-risk RS cohort [96]. As of now, the available evidence does not support the inclusion of gene expression assays into the decision-making process for RT. The abovementioned ongoing prospective trials will help to optimize the indication of RT in specific subgroups of patients [97].

### 4.3. Tumor-Response-Based Considerations

Neoadjuvant systemic treatment (NAST) is becoming more and more popular. For specific subgroups, pathological complete remission (pCR) rates are high. In initially node-positive patients undergoing NAST, the conversion to ypN0 is an important prognostic factor with a better outcome [98]. Treatment response to NAST might offer the possibility to optimize adjuvant RT in regard to indication and volumes. The most important question is the avoidance of PMRT or at least regional RT after NAST in certain circumstances. Montero et al. reviewed the available literature [99]. The median rates of loco-regional relapses in patients with ypN0 following NAST were 3.2% (range 0–7.7%) with and 24.4% (range 7.7–41.7%) without PMRT. In patients with ypN+, the corresponding numbers were 10.8% (range 0–46%) and 56.3% (range 11.2–100%) [99]. However, data are mostly derived from retrospective analyses, and there is an urgent need for prospective and randomized studies. In the RAPCHEM trial [100], patients with cT1-2cN1 were grouped into three risk categories according to the nodal status after NAST. The RT volumes were prespecified for each group. For patients who had ypN0 or pN1mi without risk factors (cT > 3 cm, G3, LVI), only whole-breast RT after BCS and no PMRT was foreseen. Despite excellent results (5 yr loco-regional recurrence rates between 2 and 3%), extrapolation of the trial results to modern practice is difficult. More than 80% of the patients had ALND (even at low risk), and protocol violations were quite common, including PMRT in >30% of patients in the low-risk group [101]. This serves as a good example that randomization is key. The phase 3 ATNEC study is ongoing: cT1-3cN1 patients with ypN0 after NAST will be randomized to additional axillary RT or ALND versus no further axillary treatment [102]. In total, 1900 patients are planned. One of the most awaited studies has been recently presented at the last San Antonio Breast Cancer Symposium: The NSABP B-51/RTOG1304 trial addresses the question of PMRT and RNI in cT1-3 cN1 that convert to ypN0 after NAST. Patients were randomized to WBI with or without regional RT after BCS and loco-regional PMRT versus no PMRT at all [103]. A total of 1556 patients with a median follow-up of 59.5 months could be analyzed. The invasive breast cancer recurrence-free interval at 5 years was similar, with 91.8% in the no RNI (59 events) and 92.7% (50 events) in the RNI arm. Of interest was an exploratory subgroup analysis regarding tumor subtype. In fact, there was a statistically significant interaction (*p* = 0.037) favoring RNI in ER/PR+/Her2- patients versus a detrimental effect for ‘more’ RT in triple-negative disease (HR 2.3; 1.00–5.25), which contains normally higher loco-regional relapse rates. Events were low, and findings might be by chance, which would call for further follow-up. In a retrospective analysis of 1966 early-stage TNBC, a higher abundance of tumor-infiltrating lymphocytes (TIL) was associated with better survival [104]. Regarding the NSABP-B51 findings, one might hypothesize that comprehensive RT fields might compromise the immune system. A very recent study demonstrated the significance of full dosimetric data, particularly the volume of low dose at 1 Gy (V1) of critical structures on lymphopenia after RT in breast cancer patients [104]. This deserves attention as especially modern RT techniques for large fields like VMAT have relatively high values (large volume) of V1 [105]. In this context, a re-analysis of the NSABP B-51 data regarding TILs and lymphopenia—if possible—would be interesting. The current NCCN guideline still strongly considers loco-regional PMRT in patients with cN+ -> ypN0 disease [29]; however, de-escalation of RT fields is an option after a case-by-case discussion with the patient. For Her2-positive disease, the NRG-BR008 (HERO) phase 3 trial evaluates the omission of RT in early-stage low-risk patients after BCS, defined by either pT1N0 or cT-3cm/cN0 with ypT0N0 after neoadjuvant chemotherapy and HER2-targeted therapy. As of 02-05-2024, accrual was 13 of 1300 patients planned [106]—still a long way to go.

## 5. Conclusions

Radiotherapy is an important pillar in the adjuvant treatment of breast cancer. Science regarding RT in breast cancer goes into two directions: escalation and de-escalation of RT. Several topics have been identified for both approaches. However, the selection of topics has no right to completeness and reflects the personal choice of the author. Less fractions and less RT volume, like PBI only, as well as technical improvements have led to a reduction in side effects and provide better and more convenient treatment. It is quite likely that in the near future five fractions in one week will be considered standard for most clinical situations in which RT is indicated. Appropriate patient selection towards low risk of relapse offers the possibility of RT omission. However, so far no subgroup of patients can be identified, who have not benefitted from adjuvant RT at all. In patients with a positive sentinel lymph node, regional RT has replaced ALND except for patients with clinical node-positive disease. Ongoing studies including biology and molecular assays should answer the question concerning which patients could safely forego irradiation and how RT volumes can be adapted. It is most likely that the response to neoadjuvant systemic treatments could help in optimization.

## Figures and Tables

**Table 1:** Important prospective studies in Luminal A (-like), ‘low-risk’ patients.

Trial	Phase 3	*n*	Main Selection Criteria	Therapy	F-Up	Recurrence
LUMINA [80]	no	500	55+ yrs; T1N0; R0 (1 mm); G1-2; Ki-67 −13.25%	ET	5 yrs	at 5 yrs: 2.3%
IDEA [81]	no	200	50–69 yrs; T1N0 R0 (2 mm); Oncotype RS: −18	ET	minimum 57 mo	overall: 4%
PRECISION [82]	no	690	50–75 yrs; T1N0; R0; G1-2; PAM50 Luminal A	ET	median 27 mo	at 2 yrs: 0.3%
PRIMETIME [83]	no	1623	60+ yrs; T1N0; R0 (1 mm); G1-2; IHC4 + C	ET	closed 03/22	n.a.
NATURAL [84]	yes	926	60+ yrs; T1N0; R0 (2 mm); G1-2	ET vs. ET + PBI	accruing	n.a.
EXPERT [75]	yes	1170	50+ yrs; T1N0; R0; G1-2; PAM50 ROR-60	ET vs. ET + WBRT	accruing	n.a.
DEBRA [85]	yes	1670	50–70 yrs; T1N0; R0; Oncotype RS −18	ET vs. ET + WBRT	accruing	n.a.
EUROPA [78]	yes	926	70+ yrs; T1N0; R0; G1-2 (G3 if T1a/b); Ki67 −20%	ET vs. RT(PBI/WBRT)	accruing	n.a. (endpoint: 2 yrs HRQoL)

Abbreviations: yrs = years; ET = endocrine treatment; PBI = partial breast irradiation only; WBRT = whole-breast radiotherapy; mo = months; F-up = follow-up; n.a. = not available; HRQoL = health-related quality of life.

## References

[1] Darby S., McGale P., Correa C., Taylor C., Arriagada R., Clarke M., Cutter D., Davies C., Ewertz M., Early Breast Cancer Trialists’ Collaborative Group (EBCTCG) (2011). Effect of radiotherapy after breast-conserving surgery on 10-year recurrence and 15-year breast cancer death: Meta-analysis of individual patient data for 10,801 women in 17 randomised trials. Lancet.

[2] Shariati S., Behroozian T., Kennedy S., Caini S., Herst P.M., Zhang L., Ding K., Karam I., van den Hurk C., Wolf J.R. (2023). Mepithel film for the prevention and treatment of acute radiation dermatitis in breast cancer: A systematic review and meta-analysis of randomized controlled trials. Support Care Cancer.

[3] Prescott R.J., Kunkler I.H., Williams L.J., King C.C., Jack W., van der Pol M., Goh T.T., Lindley R., Cairns J. (2007). A randomised controlled trial of postoperative radiotherapy following breast-conserving surgery in a minimum-risk older population. The PRIME trial. Health Technol. Assess..

[4] Velikova G., Williams L.J., Willis S., Dixon J.M., Loncaster J., Hatton M., Clarke J., Kunkler I.H., Russell N.S., MRC SUPREMO Trial UK Investigators (2018). Quality of life after postmastectomy radiotherapy in patients with intermediate-risk breast cancer (SUPREMO): 2-year follow-up results of a randomised controlled trial. Lancet Oncol..

[5] Joseph K., Vos L.J., Gabos Z., Pervez N., Chafe S., Tankel K., Warkentin H., Ghosh S., Amanie J., Powell K. (2021). Skin Toxicity in Early Breast Cancer Patients Treated with Field-In-Field Breast Intensity-Modulated Radiotherapy versus Helical Inverse Breast Intensity-Modulated Radiotherapy: Results of a Phase III Randomised Controlled Trial. Clin. Oncol. (R. Coll. Radiol.).

[6] Yee C., Wang K., Asthana R., Drost L., Lam H., Lee J., Vesprini D., Leung E., DeAngelis C., Chow E. (2018). Radiation-induced Skin Toxicity in Breast Cancer Patients: A Systematic Review of Randomized Trials. Clin. Breast Cancer.

[7] Jagsi R., Griffith K.A., Moran J.M., Ficaro E., Marsh R., Dess R.T., Chung E., Liss A.L., Hayman J.A., Mayo C.S. (2018). A Randomized Comparison of Radiation Therapy Techniques in the Management of Node-Positive Breast Cancer: Primary Outcomes Analysis. Int. J. Radiat. Oncol. Biol. Phys..

[8] Mulliez T., Veldeman L., Speleers B., Mahjoubi K., Remouchamps V., Van Greveling A., Gilsoul M., Berwouts D., Lievens Y., Van den Broecke R. (2015). Heart dose reduction by prone deep inspiration breath hold in left-sided breast irradiation. Radiother. Oncol..

[9] Mulliez T., Veldeman L., van Greveling A., Speleers B., Sadeghi S., Berwouts D., Decoster F., Vercauteren T., De Gersem W., Van den Broecke R. (2013). Hypofractionated whole breast irradiation for patients with large breasts: A randomized trial comparing prone and supine positions. Radiother. Oncol..

[10] Bekelman J.E., Lu H., Pugh S., Baker K., Berg C.D., de Gonzalez A.B., Braunstein L.Z., Bosch W., Chauhan C., Ellenberg S. (2019). Pragmatic randomised clinical trial of proton versus photon therapy for patients with non-metastatic breast cancer: The Radiotherapy Comparative Effectiveness (RadComp) Consortium trial protocol. BMJ Open.

[11] Holt F., Probert J., Darby S.C., Haviland J.S., Coles C.E., Kirby A.M., Liu Z., Dodwell D., Ntentas G., Duane F. (2023). Proton Beam Therapy for Early Breast Cancer: A Systematic Review and Meta-analysis of Clinical Outcomes. Int. J. Radiat. Oncol. Biol. Phys..

[12] Offersen B.V., Boersma L.J., Kirkove C., Hol S., Aznar M.C., Biete Sola A., Kirova Y.M., Pignol J.-P., Remouchamps V., Verhoeven K. (2015). ESTRO consensus guideline on target volume delineation for elective radiation therapy of early stage breast cancer. Radiother. Oncol..

[13] Nelms B.E., Robinson G., Markham J., Velasco K., Boyd S., Narayan S., Wheeler J., Sobczak M.L. (2012). Variation in external beam treatment plan quality: An inter-institutional study of planners and planning systems. Pract. Radiat. Oncol..

[14] Phurailatpam R., Sah M.K., Wadasadawala T., Khan A., Palottukandy J., Gayake U., Jain J., Sarin R., Pathak R., Krishnamurthy R. (2023). Can knowledge based treatment planning of VMAT for post-mastectomy locoregional radiotherapy involving internal mammary chain and supraclavicular fossa improve performance efficiency?. Front. Oncol..

[15] Bartelink H., Maingon P., Poortmans P., Weltens C., Fourquet A., Jager J., Schinagl D., Oei B., Rodenhuis C., Horiot J.C. (2015). Whole-breast irradiation with or without a boost for patients treated with breast-conserving surgery for early breast cancer: 20-year follow-up of a randomised phase 3 trial. Lancet Oncol..

[16] Hau E., Browne L., Capp A., Delaney G.P., Fox C., Kearsley J.H., Millar E., Nasser E.H., Papadatos G., Graham P.H. (2013). The impact of breast cosmetic and functional outcomes on quality of life: Long-term results from the St. George and Wollongong randomized breast boost trial. Breast Cancer Res. Treat..

[17] Coles C.E., Griffin C.L., Kirby A.M., Titley J., Agrawal R.K., Alhasso A., Bhattacharya I.S., Brunt A.M., Ciurlionis L., Chan C. (2017). Partial-breast radiotherapy after breast conservation surgery for patients with early breast cancer (UK IMPORT LOW trial): 5-year results from a multicentre, randomised, controlled, phase 3, non-inferiority trial. Lancet.

[18] www.leitlinienprogramm-onkologie.de/fileadmin/user_upload/Downloads/Leitlinien/Mammakarzinom_4_0/Version_4.4/LL_Mammakarzinom_Langversion_4.4.pdf.

[19] Haviland J.S., Owen J.R., Dewar J.A., Agrawal R.K., Barrett J., Barrett-Lee P.J., Dobbs H.J., Hopwood P., Lawton P.A., Magee B.J. (2013). The UK Standardisation of Breast Radiotherapy (START) trials of radiotherapy hypofractionation for treatment of early breast cancer: 10-year follow-up results of two randomised controlled trials. Lancet Oncol..

[20] Wang S.L., Fang H., Song Y.W., Wang W.H., Hu C., Liu Y.P., Jin J., Liu X.F., Yu Z.H., Ren H. (2019). Hypofractionated versus conventional fractionated postmastectomy radiotherapy for patients with high-risk breast cancer: A randomised, non-inferiority, open-label, phase 3 trial. Lancet Oncol..

[21] Murray Brunt A., Haviland J.S., Wheatley D.A., Sydenham M.A., Alhasso A., Bloomfield D.J., Chan C., Churn M., Cleator S., Coles C.E. (2020). Hypofractionated breast radiotherapy for 1 week versus 3 weeks (FAST-Forward): 5-year efficacy and late normal tissue effects results from a multicentre, non-inferiority, randomised, phase 3 trial. Lancet.

[22] Krug D., Baumann R., Combs S.E., Duma M.N., Dunst J., Feyer P., Fietkau R., Haase W., Harms W., Hehr T. (2021). Moderate hypofractionation remains the standard of care for whole-breast radiotherapy in breast cancer: Considerations regarding FAST and FAST-Forward. Strahlenther. Onkol..

[23] Yadav B.S., Dahiya D., Kannan P., Goyal S., Laroiya I., Irrinki S., Singh N.R., Sharma R. (2024). HYPofractionated Adjuvant RadioTherapy in 1 versus 2 weeks in high-risk patients with breast cancer (HYPART): A non-inferiority, open-label, phase III randomized trial. Trials.

[24] Meattini I., Becherini C., Boersma L., Kaidar-Person O., Marta G.N., Montero A., Offersen B.V., Aznar M.C., Belka C., Brunt A.M. (2022). European Society for Radiotherapy and Oncology Advisory Committee in Radiation Oncology Practice consensus recommendations on patient selection and dose and fractionation for external beam radiotherapy in early breast cancer. Lancet Oncol..

[25] Orecchia R., Veronesi U., Maisonneuve P., Galimberti V.E., Lazzari R., Veronesi P., Jereczek-Fossa B.A., Cattani F., Sangalli C., Luini A. (2021). ELIOT trial Intraoperative irradiation for early breast cancer (ELIOT): Long-term recurrence and survival outcomes from a single-centre, randomised, phase 3 equivalence trial. Lancet Oncol..

[26] Vaidya J.S., Bulsara M., Baum M., Wenz F., Massarut S., Pigorsch S., Alvarado M., Douek M., Saunders C., Flyger H.L. (2020). Long term survival and local control outcomes from single dose targeted intraoperative radiotherapy during lumpectomy (TARGIT-IORT) for early breast cancer: TARGIT-A randomised clinical trial. BMJ.

[27] Haussmann J., Budach W., Corradini S., Krug D., Tamaskovics B., Bölke E., Djiepmo-Njanang F.J., Simiantonakis I., Kammers K., Matuschek C. (2020). No Difference in Overall Survival and Non-Breast Cancer Deaths after Partial Breast Radiotherapy Compared to Whole Breast Radiotherapy—A Meta-Analysis of Randomized Trials. Cancers.

[28] Whelan T.J., Julian J.A., Berrang T.S., Kim D.H., Germain I., Nichol A.M., Akra M., Lavertu S., Germain F., Fyles A. (2019). External beam accelerated partial breast irradiation versus whole breast irradiation after breast conserving surgery in women with ductal carcinoma in situ and node-negative breast cancer (RAPID): A randomised controlled trial. Lancet.

[29] National Comprehensive Cancer Network. www.nccn.org/professionals/physician_gls/pdf/breast.pdf.

[30] Kunkler I.H., Williams L.J., Jack W.J.L., Cameron D.A., Dixon J.M. (2023). Breast-Conserving Surgery with or without Irradiation in Early Breast Cancer. N. Engl. J. Med..

[31] Overgaard M., Hansen P.S., Overgaard J., Rose C., Andersson M., Bach F., Kjaer M., Gadeberg C.C., Mouridsen H.T., Jensen M.B. (1997). Postoperative radiotherapy in high-risk premenopausal women with breast cancer who receive adjuvant chemotherapy. Danish Breast Cancer Cooperative Group 82b Trial. N. Engl. J. Med..

[32] Overgaard M., Jensen M.B., Overgaard J., Hansen P.S., Rose C., Andersson M., Kamby C., Kjaer M., Gadeberg C.C., Rasmussen B.B. (1999). Postoperative radiotherapy in high-risk postmenopausal breast-cancer patients given adjuvant tamoxifen: Danish Breast Cancer Cooperative Group DBCG 82c randomised trial. Lancet.

[33] Overgaard M., Nielsen H.M., Tramm T., Højris I., Grantzau T.L., Alsner J., Offersen B.V., Overgaard J., DBCG Radiotherapy Group (2022). Postmastectomy radiotherapy in high-risk breast cancer patients given adjuvant systemic therapy. A 30-year long-term report from the Danish breast cancer cooperative group DBCG 82bc trial. Radiother. Oncol..

[34] Kunkler I.H., Canney P., van Tienhoven G., Russell N.S., MRC/EORTC (BIG 2-04) SUPREMO Trial Management Group (2008). Elucidating the role of chest wall irradiation in ‘intermediate-risk’ breast cancer: The MRC/EORTC SUPREMO trial. Clin. Oncol. (R. Coll. Radiol.).

[35] Matuschek C., Nestle-Kraemling C., Haussmann J., Bölke E., Wollandt S., Speer V., Njanang F.J., Tamaskovics B., Gerber P.A., Orth K. (2019). Long-term cosmetic outcome after preoperative radio−/chemotherapy in locally advanced breast cancer patients. Strahlenther. Onkol..

[36] German Breast Group. www.gbg.de/en/trials/neorad.

[37] O’Donnell J.P.M., Murphy D., Ryan É.J., Gasior S.A., Sugrue R., O’Neill B.L., Boland M.R., Lowery A.J., Kerin M.J., McInerney N.M. (2021). Optimal reconstructive strategies in the setting of post-mastectomy radiotherapy—A systematic review and network meta-analysis. Eur. J. Surg. Oncol..

[38] Admojo L., Chidley P., Lin Y.H., Foroudi F., Jassal S., Loh S.W., Chew G., Bevington E., Ng S.L., Hyett A. (2022). Comparing Radiotherapy (RT) Late Toxicities to the Reconstructed DIEP Flap in Breast Cancer Patients Treated with Neoadjuvant RT (NART) vs. Post-Mastectomy RT (PMRT). Int. J. Radiat. Oncol. Biol. Phys..

[39] Civil Y.A., Jonker L.W., Groot Koerkamp M.P.M., Duvivier K.M., de Vries R., Oei A.L., Slotman B.J., van der Velde S., van den Bongard H.J.G.D. (2023). Preoperative Partial Breast Irradiation in Patients with Low-Risk Breast Cancer: A Systematic Review of Literature. Ann. Surg. Oncol..

[40] Meattini I., Francolini G., Di Cataldo V., Visani L., Becherini C., Scoccimarro E., Salvestrini V., Bellini C., Masi L., Doro R. (2022). Preoperative robotic radiosurgery for early breast cancer: Results of the phase II ROCK trial (NCT03520894). Clin. Transl. Radiat. Oncol..

[41] Jones E.L., Oleson J.R., Prosnitz L.R., Samulski T.V., Vujaskovic Z., Yu D., Sanders L.L., Dewhirst M.W. (2005). Randomized Trial of Hyperthermia and Radiation for Superficial Tumors. J. Clin. Oncol..

[42] Kim D.Y., Youn J.C., Park M.S., Lee S., Choi S.W., Ryu K.H., Kim L.S., Shim M.S., Lee J.J., Han S. (2019). Cardiovascular outcome of breast cancer patients with concomitant radiotherapy and chemotherapy: A 10-year multicenter cohort study. J. Cardiol..

[43] Montero A., Ciérvide R. (2022). Preoperative Radio(Chemo)Therapy in Breast Cancer: Time to Switch the Perspective?. Curr. Oncol..

[44] Breast Cancer Study of Preoperative Pembrolizumab + Radiation—Full Text View—ClinicalTrials.gov. www.clinicaltrials.gov.

[45] Neo-Adjuvant Chemotherapy Combined with Stereotactic Body Radiotherapy to the Primary Tumour +/− Durvalumab, +/− Oleclumab in Luminal B Breast Cancer. www.clinicaltrials.gov.

[46] Converting HR+ Breast Cancer into an Individualized Vaccine. www.clinicaltrials.gov.

[47] Meattini I., Becherini C., Caini S., Coles C.E., Cortes J., Curigliano G., de Azambuja E., Isacke C.M., Harbeck N., Kaidar-Person O. (2024). International multidisciplinary consensus on the integration of radiotherapy with new systemic treatments for breast cancer: European Society for Radiotherapy and Oncology (ESTRO)-endorsed recommendations. Lancet Oncol..

[48] Koide Y., Nagai N., Adachi S., Ito M., Kawamura M., Ito M., Ito F., Shindo Y., Aoyama T., Shimizu H. (2024). Impact of concurrent antibody-drug conjugates and radiotherapy on symptomatic radiation necrosis in breast cancer patients with brain metastases: A multicenter retrospective study. J. Neurooncol..

[49] Chua B.H., Link E.K., Kunkler I.H., Whelan T.J., Westenberg A.H., Gruber G., Bryant G., Ahern V., Purohit K., Graham P.H. (2022). BIG 3–07/TROG 07.01 trial investigators. Radiation doses and fractionation schedules in non-low-risk ductal carcinoma in situ in the breast (BIG 3-07/TROG 07.01): A randomised, factorial, multicentre, open-label, phase 3 study. Lancet.

[50] Bosma S., van Werkhoven E., Bartelink H., Fourquet A., Hurkmans C., Maduro J., Rutgers E., Scheijmans L., Schinagl D., Stam M. (2024). Young boost randomized phase III trial of high vs. low boost radiation in young breast cancer patients: 10-years results. Eur. J. Cancer.

[51] Schmitt M., Menoux I., Chambrelant I., Hild C., Petit T., Mathelin C., Noël G. (2022). Adjuvant hypofractionated radiotherapy with simultaneous integrated boost after breast-conserving surgery: A systematic literature review. Transl. Oncol..

[52] Coles C.E., Haviland J.S., Kirby A.M., Griffin C.L., Sydenham M.A., Titley J.C., Bhattacharya I., Brunt A.M., Chan H.Y.C., Donovan E.M. (2023). Dose-escalated simultaneous integrated boost radiotherapy in early breast cancer (IMPORT HIGH): A multicentre, phase 3, non-inferiority, open-label, randomised controlled trial. Lancet.

[53] Dunst J., Krug D., Schreiber A., Boicev A.D., Zimmer J., Laubach R., Weidner N., Dinges S.E., Hipp M., Schneider R. (2020). Patient Reported Experience with Treatment Modalities and Safety of Adjuvant Breast Radiotherapy—First Results of the Randomized HYPOSIB—Study. Int. J. Radiat. Oncol. Biol. Phys..

[54] Vicini F.A., Winter K., Freedman G.M., Arthur D.W., Hayman J.A., Rosenstein B.S., Bentzen S.M., Li A., Lyons J., Tomberlin J.K. (2022). NRG RTOG 1005: A phase III trial of hypo fractionated whole breast irradiation with concurrent boost vs. Conventional whole breast irradiation plus sequential boost following lumpectomy for high risk early-stage breast cancer. Int. J. Radiat. Oncol. Biol. Phys..

[55] Garcia Zanuguera C., Gadea Quintero J., Curbelo Artiles A.G., Mateu Castell L., Maturana J.E.M., Ortiz Gonzalez I., Alastuey I., Pardo J. (2022). Safety and feasibility of simultaneous integrated boost in extreme 1-week hypofractionated radiotherapy for early breast cancer. Int. J. Radiat. Oncol. Biol. Phys..

[56] Chatterjee S., Chakraborty S. (2020). Hypofractionated radiation therapy comparing a standard radiotherapy schedule (over 3 weeks) with a novel 1-week schedule in adjuvant breast cancer: An open-label randomized controlled study (HYPORT-Adjuvant)-study protocol for a multicentre, randomized phase III trial. Trials.

[57] Hypofractionated LocoRegional Radiotherapy in Breast Cancer (RHEAL). https://clinicaltrials.gov/study/NCT04228991.

[58] Targit. www.targit.org.uk.

[59] Vos E.L., Siesling S., Baaijens M.H.A., Verhoef C., Jager A., Voogd A.C., Koppert L.B. (2017). Omitting re-excision for focally positive margins after breast-conserving surgery does not impair disease-free and overall survival. Breast Cancer Res. Treat..

[60] Budach W., Bölke E., Kammers K., Gerber P.A., Nestle-Krämling C., Matuschek C. (2015). Adjuvant radiation therapy of regional lymph nodes in breast cancer—A meta-analysis of randomized trials—An update. Radiat. Oncol..

[61] Early Breast Cancer Trialists’ Collaborative Group (EBCTCG) (2023). Radiotherapy to regional nodes in early breast cancer: An individual patient data meta-analysis of 14 324 women in 16 trials. Lancet.

[62] Haussmann J., Budach W., Tamaskovics B., Bölke E., Corradini S., Djiepmo-Njanang F.J., Kammers K., Matuschek C. (2019). Which target volume should be considered when irradiating the regional nodes in breast cancer? Results of a network-meta-analysis. Radiat. Oncol..

[63] Kim Y.B., Byun H.K., Kim D.Y., Ahn S.J., Lee H.S., Park W., Kim S.S., Kim J.H., Lee K.C., Lee I.J. (2022). Effect of Elective Internal Mammary Node Irradiation on Disease-Free Survival in Women with Node-Positive Breast Cancer: A Randomized Phase 3 Clinical Trial. JAMA Oncol..

[64] Kaidar-Person O., Fortpied C., Hol S., Weltens C., Kirkove C., Budach V., Peignaux-Casasnovas K., van der Leij F., Vonk E., Valli M. (2022). The association of internal mammary and medial supraclavicular lymph node radiation technique with clinical outcomes: Results from the EORTC 22922/10925 randomised trial. Radiother. Oncol..

[65] Giuliano A.E., Ballman K.V., McCall L., Beitsch P.D., Brennan M.B., Kelemen P.R., Ollila D.W., Hansen N.M., Whitworth P.W., Blumencranz P.W. (2017). Effect of Axillary Dissection vs. No Axillary Dissection on 10-Year Overall Survival among Women with Invasive Breast Cancer and Sentinel Node Metastasis: The ACOSOG Z0011 (Alliance) Randomized Clinical Trial. JAMA.

[66] Bartels S.A.L., Donker M., Poncet C., Sauvé N., Straver M.E., van de Velde C.J.H., Mansel R.E., Blanken C., Orzalesi L., Klinkenbijl J.H.G. (2023). Radiotherapy or Surgery of the Axilla after a Positive Sentinel Node in Breast Cancer: 10-Year Results of the Randomized Controlled EORTC 10981-22023 AMAROS Trial. J. Clin. Oncol..

[67] Sávolt Á., Péley G., Polgár C., Udvarhelyi N., Rubovszky G., Kovács E., Győrffy B., Kásler M., Mátrai Z. (2017). Eight-year follow up result of the OTOASOR trial: The Optimal Treatment of the Axilla–Surgery or Radiotherapy after positive sentinel lymph node biopsy in early-stage breast cancer: A randomized, single centre, phase III, non-inferiority trial. Eur. J. Surg. Oncol..

[68] Haussmann J., Matuschek C., Bölke E., Orth K., Ghadjar P., Budach W. (2019). The Role of Local Treatment in Oligometastatic and Oligoprogressive Cancer. Dtsch. Arztebl. Int..

[69] Chmura S.J., Winter K.A., Woodward W.A., Borges V.F., Salama J.K., Al-Hallaq H.A., Matuszak M., Milano M.T., Jaskowiak N.T., Bandos H. (2022). NRG-BR002: A phase IIR/III trial of standard of care systemic therapy with or without stereotactic body radiotherapy (SBRT) and/or surgical resection (SR) for newly oligometastatic breast cancer (NCT02364557). J. Clin. Oncol..

[70] Merloni F., Palleschi M., Casadei C., Romeo A., Curcio A., Casadei R., Stella F., Ercolani G., Gianni C., Sirico M. (2023). Oligometastatic breast cancer and metastasis-directed treatment: An aggressive multimodal approach to reach the cure. Ther. Adv. Med. Oncol..

[71] Ng J., Pennell R., Formenti S.C. (2022). The initial experience of MRI-guided precision prone breast irradiation with daily adaptive planning in treating early stage breast cancer patients. Front. Oncol..

[72] Liu X., Li Z., Yin Y. (2023). Clinical application of MR-Linac in tumor radiotherapy: A systematic review. Radiat. Oncol..

[73] Matuschek C., Bolke E., Haussmann J., Mohrmann S., Nestle-Kramling C., Gerber P.A., Corradini S., Orth K., Kammers K., Budach W. (2017). The benefit of adjuvant radiotherapy after breast conserving surgery in older patients with low risk breast cancer—A meta-analysis of randomized trials. Radiat. Oncol..

[74] Sjöström M., Chang S.L., Fishbane N., Davicioni E., Zhao S.G., Hartman L., Holmberg E., Feng F.Y., Speers C.W., Pierce L.J. (2019). Clinicogenomic Radiotherapy Classifier Predicting the Need for Intensified Locoregional Treatment after Breast-Conserving Surgery for Early-Stage Breast Cancer. J. Clin. Oncol..

[75] Examining Personalised Radiation Therapy for Low-Risk Early Breast Cancer (EXPERT), NCT02889874. NCT02889874.

[76] Savard M.F., Alzahrani M.J., Saunders D., Chang L., Arnaout A., Ng T.L., Brackstone M., Vandermeer L., Hsu T., Awan A.A. (2021). Experiences and Perceptions of Older Adults with Lower-Risk Hormone Receptor-Positive Breast Cancer about Adjuvant Radiotherapy and Endocrine Therapy: A Patient Survey. Curr. Oncol..

[77] Morris A., Hanes D.A., Kaplan H. (2023). Long Term Outcomes of Radiation (RT)-Monotherapy vs. Combined RT + Endocrine Therapy (RT+ET) in Low-Risk Early-Stage Breast Cancer Patients 70 Years or Older after Breast-Conserving Surgery (BCS). Int. J. Radiat. Oncol. Biol. Phys..

[78] Meattini I., Poortmans P.M.P., Marrazzo L., Desideri I., Brain E., Hamaker M., Lambertini M., Miccinesi G., Russell N., Saieva C. (2021). Exclusive endocrine therapy or partial breast irradiation for women aged ≥ 70 years with luminal A-like early stage breast cancer (NCT04134598-EUROPA): Proof of concept of a randomized controlled trial comparing health related quality of life by patient reported outcome measures. J. Geriatr. Oncol..

[79] Mann G.B., Skandarajah A.R., Zdenkowski N., Hughes J., Park A., Petrie D., Saxby K., Grimmond S.M., Murugasu A., Spillane A.J. (2024). Postoperative radiotherapy omission in selected patients with early breast cancer following preoperative breast MRI (PROSPECT): Primary results of a prospective two-arm study. Lancet.

[80] Whelan T.J., Smith S., Parpia S., Fyles A.W., Bane A., Liu F.F., Rakovitch E., Chang L., Stevens C., Bowen J. (2023). Omitting Radiotherapy after Breast-Conserving Surgery in Luminal A Breast Cancer. N. Engl. J. Med..

[81] Jagsi R., Griffith K.A., Harris E.E., Wright J.L., Recht A., Taghian A.G., Lee L., Moran M.S., Small W., Johnstone C. (2024). Omission of Radiotherapy after Breast-Conserving Surgery for Women with Breast Cancer with Low Clinical and Genomic Risk: 5-Year Outcomes of IDEA. J. Clin. Oncol..

[82] Braunstein L.Z., Wong J., Dillon D.A., Chen Y.H., Catalano P., Cahlon O., El-Tamer M.B., Jimenez R., Khan A., Perez C. (2023). Preliminary report of the PRECISION Trial (Profiling Early Breast Cancer for Radiotherapy Omission): A Phase II Study of Breast-Conserving Surgery without Adjuvant Radiotherapy for Favorable-Risk Breast Cancer [abstract]. Proceedings of the 2022 San Antonio Breast Cancer Symposium.

[83] Kirwan C.C., Coles C.E., Bliss J., Kirwan C., Kilburn L., Fox L., Cheang M., Griffin C., Francis A., Kirby A. (2016). It’s PRIMETIME. Postoperative Avoidance of Radiotherapy: Biomarker Selection of Women at Very Low Risk of Local Recurrence. Clin. Oncol..

[84] Offersen B., Al-Rawi S., Bechmann T., Kamby C., Mathiessen L., Nielsen H., Nielsen M., Stenbygaard L., Jensen M., Alsner J. The DBCG RT NATURAL trial: Accelerated partial breast irradiation versus no irradiation for early stage breast cancer, a clinically controlled randomized phase III trial. Proceedings of the Danske Kræftforskningsdage.

[85] White J.R., Anderson S.J., Harris E.E., Mamounas E.P., Stover D.G., Ganz P.A., Jagsi R., Cecchini R.S., Bergom C., Theberge V. (2022). NRG-BR007: A phase III trial evaluating de-escalation of breast radiation (DEBRA) following breast-conserving surgery (BCS) of stage 1, hormone receptor1, HER2-, RS 18 breast cancer. J. Clin. Oncol..

[86] Jagsi R., Chadha M., Moni J., Ballman K., Laurie F., Buchholz T.A., Giuliano A., Haffty B.G. (2014). Radiation field design in the ACOSOG Z0011 (Alliance) Trial. J. Clin. Oncol..

[87] Katz M.S., McCall L., Ballman K., Jagsi R., Haffty B.G., Giuliano A.E. (2020). Nomogram-based estimate of axillary nodal involvement in ACOSOG Z0011 (Alliance): Validation and association with radiation protocol variations. Breast Cancer Res. Treat..

[88] Henke G., Knauer M., Ribi K., Hayoz S., Gérard M.A., Ruhstaller T., Zwahlen D.R., Muenst S., Ackerknecht M., Hawle H. (2018). Tailored axillary surgery with or without axillary lymph node dissection followed by radiotherapy in patients with clinically node-positive breast cancer (TAXIS): Study protocol for a multicenter, randomized phase-III trial. Trials.

[89] Algara López M., Rodríguez García E., Beato Tortajada I., Martínez Arcelus F.J., Salinas Ramos J., Rodríguez Garrido J.R., Sanz Latiesas X., Soler Rodríguez A., Juan Rijo G., Flaquer García A. (2020). OPTimizing Irradiation through Molecular Assessment of Lymph node (OPTIMAL): A randomized open label trial. Radiat. Oncol..

[90] Tinterri C., Gentile D., Gatzemeier W., Sagona A., Barbieri E., Testori A., Errico V., Bottini A., Marrazzo E., Dani C. (2022). Preservation of Axillary Lymph Nodes Compared with Complete Dissection in T1-2 Breast Cancer Patients Presenting One or Two Metastatic Sentinel Lymph Nodes: The SINODAR-ONE Multicenter Randomized Clinical Trial. Ann. Surg. Oncol..

[91] de Boniface J., Filtenborg Tvedskov T., Rydén L., Szulkin R., Reimer T., Kuehn T., Kontos M., Gentilini O.D., Olofsson Bagge R., Sund M. (2024). Omitting Axillary Dissection in Breast Cancer with Sentinel-Node Metastases. N. Engl. J. Med..

[92] de Wild S.R., van Roozendaal L.M., de Wilt J.H.W., van Dalen T., van der Hage J.A., van Duijnhoven F.H., Simons J.M., Schipper R.J., de Munck L., van Kuijk S.M.J. (2024). De-escalation of axillary treatment in the event of a positive sentinel lymph node biopsy in cT1-2 N0 breast cancer treated with mastectomy: Nationwide registry study (BOOG 2013-07). Br. J. Surg..

[93] Alkner S., de Boniface J., Lundstedt D., Mjaaland I., Ryden L., Vikstrom J., Bendahl P.O., Holmberg E., Sackey H., Wieslander E. (2023). Protocol for the T-REX-trial: Tailored regional external beam radiotherapy in clinically node-negative breast cancer patients with 1-2 sentinel node macrometastases—An open, multicentre, randomised non-inferiority phase 3 trial. BMJ Open.

[94] Regional Radiotherapy in Biomarker Low-Risk Node Positive and T3N0 Breast Cancer. https://clinicaltrials.gov/study/NCT03488693.

[95] Qi W.X., Cao L., Zheng S., Xu C., Cai R., Xu H., Cai G., Chen J. (2022). IMNI PRECISION trial protocol: A phase II, open-label, non-inferior randomized controlled trial of tailoring omission of internal mammary node irradiation for early-stage breast cancer. BMC Cancer.

[96] Xie S.J., Wang R.J., Wu S.G., Zhang F.X. (2024). 21-gene recurrence score in predicting the outcome of postoperative radiotherapy in T1-2N1 luminal breast cancer after breast-conserving surgery. Breast.

[97] Krug D., Baumann R., Budach W., Duma M.N., Dunst J., Feyer P., Fietkau R., Haase W., Harms W., Hehr T. (2020). Commercially Available Gene Expression Assays as Predictive Tools for Adjuvant Radiotherapy? A Critical Review. Breast Care.

[98] Gerber B., Schneeweiss A., Möbus V., Golatta M., Tesch H., Krug D., Hanusch C., Denkert C., Lübbe K., Heil J. (2022). Pathological Response in the Breast and Axillary Lymph Nodes after Neoadjuvant Systemic Treatment in Patients with Initially Node-Positive Breast Cancer Correlates with Disease Free Survival: An Exploratory Analysis of the GeparOcto Trial. Cancers.

[99] Montero Á., Ciérvide R., Poortmans P. (2019). When Can We Avoid Postmastectomy Radiation Following Primary Systemic Therapy?. Curr. Oncol. Rep..

[100] de Wild S.R., de Munck L., Simons J.M., Verloop J., van Dalen T., Elkhuizen P.H.M., Houben R.M., van Leeuwen A.E., Linn S.C., Pijnappel R.M. (2022). De-escalation of radiotherapy after primary chemotherapy in cT1-2N1 breast cancer (RAPCHEM; BOOG 2010-03): 5-year follow-up results of a Dutch, prospective, registry study. Lancet Oncol..

[101] Boersma L.J., Verloop J., Voogd A.C., Elkhuizen P.H.M., Houben R., van Leeuwen A.E., Linn S., de Munck L., Pijnappel R., Strobbe L. (2020). Radiotherapy after primary CHEMotherapy (RAPCHEM): Practice variation in a Dutch registration study (BOOG 2010-03). Radiother. Oncol..

[102] Goyal A., Cramp S., Marshall A., Wheatley D., Hammonds N., Puri S., Homer T., Vale L., Butt R., Mir R. (2022). Abstract OT1-04-01: ATNEC: A Multi-Centre, Randomised Trial Investigating Whether Axillary Treatment Can Be Avoided in T1-3N1M0 Breast Cancer Patients with No Residual Cancer in the Lymph Glands after Neoadjuvant Chemotherapy (Clinicaltrials.Gov: Nct04109079). Cancer Res..

[103] Mamounas E.P. GS02-07 Loco-Regional Irradiation in Patients with Biopsy-Proven Axillary Node Involvement at Presentation Who Become Pathologically Node-Negative after Neoadjuvant Chemotherapy: Primary Outcomes of NRG Oncology/NSABP B-51/RTOG 1304. Proceedings of the 2023 San Antonio Breast Cancer Symposium.

[104] Leon-Ferre R.A., Jonas S.F., Salgado R., Loi S., de Jong V., Carter J.M., Nielsen T.O., Leung S., Riaz N., Chia S. (2024). Tumor-Infiltrating Lymphocytes in Triple-Negative Breast Cancer. JAMA.

[105] Chen F., Zhou P., Ren G., Lee E.K.W., Liu Q., Shen Y., Wang Y., El Helali A., Jin J.Y., Fu P. (2024). Interpretable deep learning insights: Unveiling the role of 1 Gy volume on lymphopenia after radiotherapy in breast cancer. Radiother. Oncol..

[106] NRG-BR008 (“HERO”): A Phase III Randomized Trial Seeking to Optimize Use of Radiotherapy in Patients with Early-Stage, Low Risk, HER2-Positive Breast Cancer. https://www.nrgoncology.org.

